# Supplementary material for: Liquid metal interface enables glassy MOF membranes with defect-mediated CO₂ transport
Source: Nat Commun. 2025 Oct 28;16:9510. doi: 10.1038/s41467-025-64583-9 (PMC12568993; doi:10.1038/s41467-025-64583-9)
Supplement: Supplementary file 1 — Supporting Information [file 41467_2025_64583_MOESM1_ESM.pdf]

**Supplementary Information**

# Liquid Metal Interface Enables Glassy MOF Membranes with Defect-Mediated $CO_2$ Transport

Xiaoheng Jin[1], Xing Wu[1], Derrick Ng[1], Aaron W. Thornton[1], Durga Acharya[1], Huanting Wang [2], Zongli Xie[1*]

[1]CSIRO Manufacturing, Private Bag 10, Clayton, VIC 3168, Australia.

[2]Department of Chemical and Biological Engineering, Monash University, Clayton, VIC 3168, Australia

E-mail: zongli.xie@csiro.au

**This file contains Supplementary Figures, Tables, Methods, and References supporting the findings of the main article.**

**Supporting Text**

**Effect of Synthesis Conditions, ZIF-zni impurity, and membrane formation**

Previous studies[1,2] have shown that inhomogeneous mixing of benzimidazole (bIm) and imidazole (Im) precursors lead to zni-phase crystallization in ZIF-62. However, the impact of synthesis condition on glassy membrane formation and performance remains largely unexplored. Here, we demonstrate that the concentration of ZIF-zni impurity phase, as calculated from XRD patterns, is highly dependent on the precursor mixing order (Figure S1, A–F). The highest purity (99.7%) is obtained by protocol 1 which consists of first mixing bIm and $nNO_3$, followed by Im addition. Most widely reported method involved mixing bIm and Im first, followed by the addition of $Zn(NO_3)_2$, resulting in a purity of 54%.

Table S1. Six permutations for mixing three precursors for solvothermal synthesis of ZIF-62 and result ZIF-zni impurity phase in the washed crystals.

| Precursor | P1 | P2 | P3 | P4 | P5 | P6 |
|---|---|---|---|---|---|---|
| $Zn(NO_3)_2.6H_2O$ | 3 | 3 | 2 | 2 | 1 | 1 |
| bIm | 1 | 2 | 1 | 3 | 2 | 3 |
| Im | 2 | 1 | 3 | 1 | 3 | 2 |
| Result ZIF-zni impurity | 0.5 ± 0.3% | 15.2 ± 2.5% | 26.1 ± 2.1% | 33.6 ± 2.9% | 54.1 ± 2.5% | 56.2 ± 3.2% |

The relative phase composition of ZIF-62 and ZIF-zni in the P1–6 sample was estimated via Rietveld refinement of PXRD data using the GSAS-II software package. A two-phase model was adopted, employing crystallographic information files (CIFs) for ZIF-62 and ZIF-zni sourced from Nozari et al. (J. Chem. Phys., 2020, DOI: 10.1063/5.0031941) and Chokbunpiam et al. (Microporous Mesoporous Mater., 2013, DOI: 10.1016/j.micromeso.2012.12.047), respectively.

Refinement parameters included background correction, zero shift, peak profile, and independent scale factors for each phase. The relative weight fractions were calculated using the following equation:

$$W_i = \frac{S_i Z_i M_i}{\sum_j S_j Z_j M_j}$$

Where $S_i$ is the refined scale factor taken from the best fitting by GSAS-II program, $Z_i$ is the number of formulat units per unit cell, and $M_i$ is the molecular weight of phase i.

The concentration ZIF-zni significantly affects membrane formation. Despite extensive solvent exchange with methanol and subsequent drying at 150°C, high impurity samples yield highly defective membranes, whereas low impurity samples form smooth, defect-free membranes. Notably, the melting temperatures of high- and low-impurities samples are similar, suggesting that defect formation during melt quenching of high impurity crystals arises from phase separation in the molten state.

Bubble formation or even explosion during ZIF-62 melting has been attributed to partial linker decomposition. However, $^1$H NMR analysis of synthesized, activated, and digested (by deuterium

chloride) glassy ZIF-62 membranes reveals no detectable change of ligand ratio. Instead, we identify incomplete DMF removal as the primary cause for the formation or even explosive burst of bubbles. The high surface tension of the MOF liquid and slow diffusion of gaseous DMF lead to trapped gas pockets, resulting in explosive rupture at high temperatures. Complete DMF removal, achievable by three methanol washes (20 mL each), as confirmed by $^1$H NMR, was enough to remove all the trapped DMF.

**Effect of liquid metal bath composition and effect on membrane formation.**

Gallium and iridium form a eutectic alloy when the iridium content exceeds 25 wt%[3]. In this study, the liquid metal bath consists of 200-500 mg of gallium and four layers of sputtered iridium (~60 nm thick) on a glass slide. Because gallium does not alloy with iridium, the bath can be regarded as pure gallium. To explore more cost-effective alternatives, tin was also used as a bath. Tin is commonly employed in glass fabrication to produce smooth surfaces due to its ability to form a uniform, defect-free contact with molten glass. Our experiments confirmed that glassy MOF also forms on liquid tin. However, its high surface tension causes it to form droplet on the glass slide, bending the MOF membrane. To compensate the surface tension of liquid tin, a tin foil was used to fabricate glassy MOF membrane. The low thickness of tin foil compensates the curvature formation of the cooled droplet, however, the flatness of glassy MOF membrane made from tin is not ideal for gas permeation test.

Other types of liquid metal, such as gallium-tin (tin 10%) and gallium-indium (Gallistan, indium 25%) were also used for the fabrication of agZIF-62 membranes.

**Table S2.** Surface Tension of Liquid Metals and Ga–Sn Alloys at Melting Point and Elevated Temperature (723 K).

| Composition | Surface Tension (Melting Point) mN/m | Surface Tension (723K) mN/m | Note | Ref |
|---|---|---|---|---|
| Gallium | 700–750, 29.8 °C | 542-580 | Oxide skin surface tension ~360mN/m | [4] |
| Tin | 559–569, 231.5 °C | 540–550 | - | [5,6] |
| Gallistan | 524-545, 10.5 °C | ~548.8 | Surface tension is not highly sensitive to temperature | [7,8] |
| Ga-Sn (10%) | ~620–640, 20.0 °C | - | Oxidizes rapidly; alters surface tension and composition. | [9] |

**Effect of Heat Treatment Processes in membrane performance.**

Previous studies have shown that the ramping rate has minimal influence on the vitrification of ZIF-62, with both 10 K/min and 50 K/min leading to complete glass transformation of the crystalline phase[10]. However, the effects of soaking conditions remain debated and seem to depend on factors such as fabrication method, furnace temperature profile, and other variables. Reports indicate that prolonged soaking at the melting point of ZIF-62 (beyond 10 minutes) may lead to partial decomposition, resulting in bubble formation and increased sample opacity. Based on this observation, Yuanzheng Yue et al. proposed that a short soaking time of 1 minute is sufficient to form defect-free glassy MOFs[10]. In contrast, other studies successfully fabricated millimetre-sized, bubble-

free glassy MOF particles using extended soaking times (>30 minutes) under vacuum hot-pressing conditions[11].

In the previous section, we attributed bubble formation to residual DMF trapped within the crystal structure due to incomplete activation, acting as an explosive agent during melting. Considering these factors, we opted for a 30-minute soaking period to ensure complete membrane formation and minimize defects.

Quenching has traditionally been considered essential for glass formation in ZIF-62. However, the impact of other thermal steps, such as tempering and annealing, on membrane formation is less understood and insufficiently explored. To address this gap, we conducted a series of experiments evaluating the effects of different thermal processing routes. A key finding is that due to the high configurational freedom of the zinc–imidazolate framework, all four thermal treatments tested lead to the formation of glassy ZIF-62. Recrystallization appears to require significantly longer timescales than those typically involved in heat treatment. Therefore, while thermal history does not influence the glass formation itself, it does have a measurable impact on gas permeation performance. The specific heat treatment conditions and their impact on resulting membrane performance are summarized in the table below.

**Table S3.** Effect of heat treatment process in $a_g$ZIF-62 membrane performance.

| Heat Treatment Processes | Program | $H_2$ Permeance (GPU) | $CO_2$ Permeance (GPU) | $N_2$ Permeance (GPU) | $CO_2/N_2$ Selectivity |
|---|---|---|---|---|---|
| Melt-Normalizing | Natural cooling in UHP $N_2$. | 295.9 ± 12.2 | 1621.4 ± 23.1 | 21.82 ± 1.4 | 74.32 ± 5.1 |
| Melt-Annealing | Cooling rate 0.5 K/min in UHP $N_2$. | 310.4 ± 11.8 | 1675.2 ± 24.7 | 22.95 ± 1.3 | 72.99 ± 4.8 |
| Melt-Gas Quenching | Cooling with 300ml/min UHP $N_2$ for 20 min with gas tube chilled by cold water. | 295.2 ± 5.9 | 1589.7 ± 21.9 | 20.63 ± 1.2 | 77.08 ± 5.2 |
| Melt-Cooling-Tempering | Gas quenching followed by ramping up to Tg at 10 K/min and then natural cooling. | 299.3 ± 13.0 | 1638.8 ± 22.5 | 21.17 ± 1.5 | 77.38 ± 5.7 |

**Effect of particle size of crystalline ZIF-62 in membrane performance.**

The effect of crystalline ZIF-62 particle size on membrane performance was examined by sieving as-synthesized activated ZIF-62 crystals through stainless steel mesh. Two mesh sizes of 1200 and 2500 were used, corresponding to particle size of 5 and 12 microns, respectively. Regardless of particle size, all membranes were prepared using the same procedure described in the main text, resulting in glassy ZIF-62 membranes with similar thickness and diameter. The permeation test was conducted with single gas, in a constant-volume, variable-pressure rig at room temperature and 1 bar.

**Table S4.** Effect of particle size in membrane performance.

| Particle size | $H_2$ Permeance | $CO_2$ Permeance | $N_2$ Permeance | $CO_2/N_2$ Selectivity |
|---|---|---|---|---|
| < 5 (> 2500 Mesh) | 295.9 ± 12.2 | 1621.4 ± 23.1 | 21.82 ± 1.4 | 74.3 ± 4.9 |
| 5-12 (1200-2500 Mesh) | 301.7 ± 10.9 | 1631.1 ± 15.4 | 20.88 ± 2.1 | 78.1 ± 7.8 |
| > 15 (< 1200 Mesh) | 1296.4 ± 10.5 | 1649.5 ± 15.7 | 19.93 ± 1.2 | 82.8 ± 5.0 |

**Modelling Summary**

A mathematical model was developed to elucidate the underlying transport mechanisms governing separation performance. Permeability ($P$) is a product of solubility ($S$) and diffusivity ($D$), expressed as,

$$P = SD$$

Solubility in polymer membranes has been shown to be a function of potential energy ($\epsilon$), also known as well depth, of the gas molecule[12]. This can also be the case for inorganic membranes such as silicas, zeolites and MOFs. However, these materials also exhibit adsorption properties that depend on pore size, shape and functionality. Therefore, solubility in glassy ZIFs can be described as a combination of these mechanisms, called solubility and adsorption, respectively, expressed as,

$$S = \exp\left(\frac{\alpha\epsilon}{RT}\right) + \beta C_{Ads}(\sigma, \epsilon, d, T)$$

where $C_{Ads}$ is the concentration of gas in adsorbed phase that depends on kinetic diameter ($\sigma$), potential energy ($\epsilon$), average pore size ($d$) and temperature ($T$), $R$ is the universal gas constant and, $\alpha$ and $\beta$ are constants. There are many models to predict adsorption including Langmuir, Freundlich, Temkin, and multi-layer models. In this study, the Topologically Integrated Mathematical Thermodynamic Adsorption Model (TIMTAM) derived from mean field theory has been adopted. TIMTAM offers an analytical approach (rather than numerical or simulation-based) and incorporates pore sizes and shapes[13].

Diffusivity in polymer membranes has been shown to be a function of the square of kinetic diameter ($\sigma^2$), as the rate of transport depends on chain opening events that allows the gas molecules to hop through the membrane[12]. This is also applicable to inorganic membranes; however, it is crucial to incorporate an activated Knudsen component as most pores are permanently open. Therefore, diffusivity will depend on both molecular sieving (related to molecular size) and velocity (related to molecular mass), as follows:

$$D = \exp\left(-\frac{\gamma\sigma^2}{RT}\right) + \delta\sqrt{\frac{8RT}{\pi m}}$$

where $m$ is molecular mass, and $\gamma$ and $\delta$ are constants.

The model was fitted to the experimental data using a least-square algorithm in MATLAB version R2020a (lsqcurvefit) based on the adjustable constants. Figure 2A provides a breakdown of the

contributions from each transport mechanism. Molecular sieving clearly depends on kinetic diameter whereas diffusivity rapidly decreases with increasing kinetic diameter. On the other hand, adsorption depends on the potential energy and kinetic diameter of the gas molecule. $CO_2$ has the highest adsorption due to strong interactions and low kinetic diameter suitable for the ZIF structure. Solubility favors the gases with the highest potential energy, $C_2H_4$ followed by $SF_6$, followed by $CO_2$ and others whereas Knudsen diffusion favours the lightest gases, $H_2$ followed by $CH_4$ and so on. During the fitting process, a balanced contribution of each mechanism was found to yield the best data fit. Solubility and adsorption emerge as the most dominant mechanisms, as indicated by the highest permeability observed for $CO_2$. Molecular sieving plays a secondary yet significant mechanism, explaining the sustrained high permeability of $H_2$. Knudsen diffusion, while the least important mechanism, remains necessary to explain to account for the observed permeability properties.

## Supporting Figures

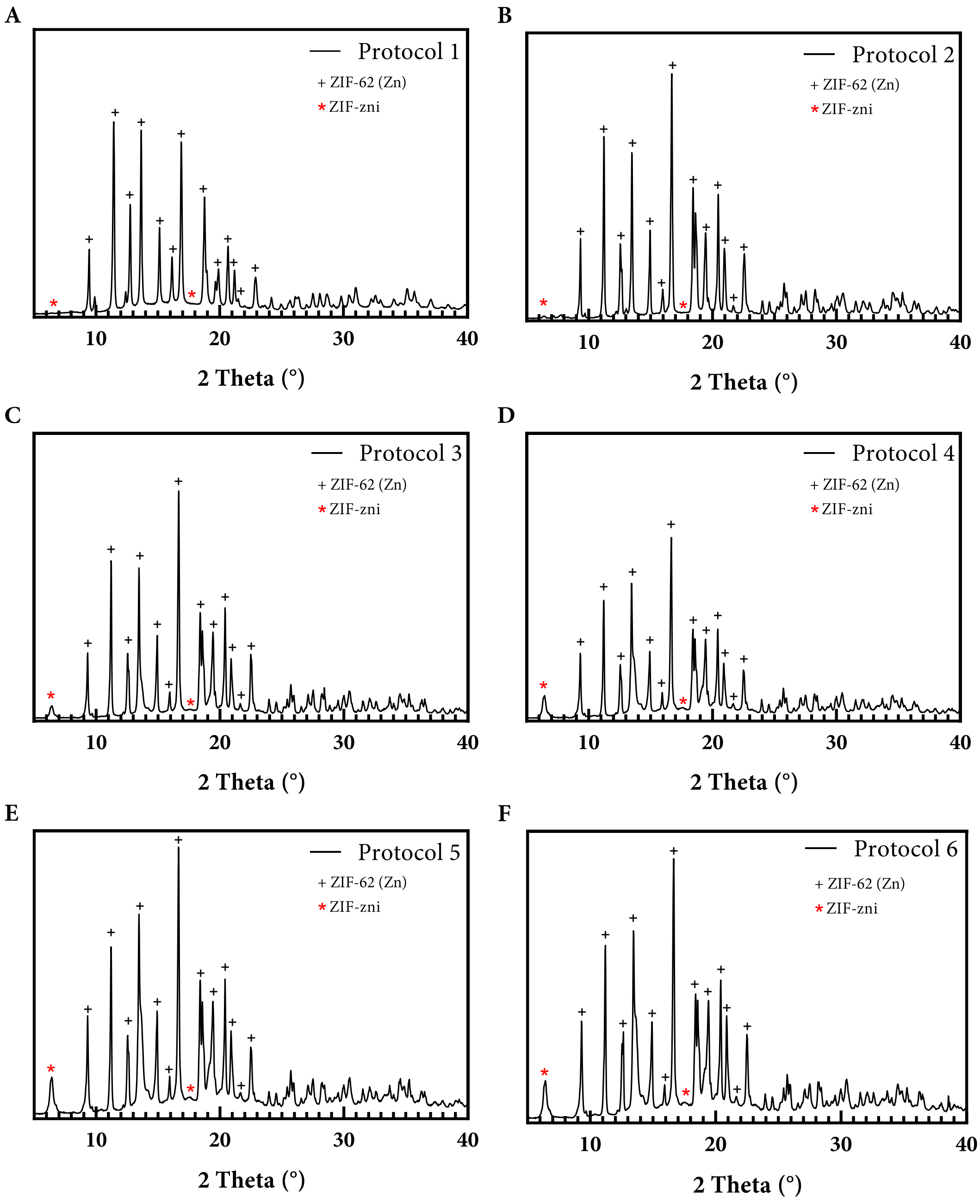


**Figure S1.** The XRD patterns of as-synthesized ZIF-62 crystals prepared via Protocols 1–6. Reflections marked with (+) correspond to ZIF-62, indexed to the Pbca space group. Peaks marked with (*) indicate the presence of ZIF-zni, indexed to the I$\bar{4}$3m space group, with the (110) and (330) facets appearing at $2\theta = 5.6°$ and 16.5°, respectively.

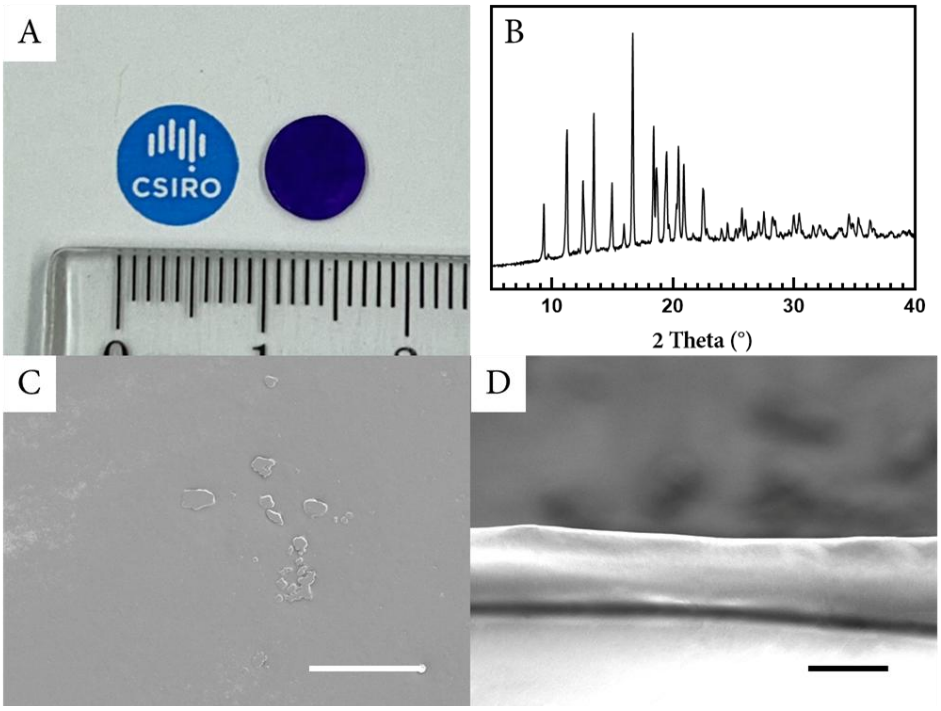


**Figure S2.** The $a_g$ZIF-62 (Co) membrane fabricated using liquid metal (Ga) bath. A: Digital image of $a_g$ZIF-62 (Co) membrane. B: XRD pattern of as-synthesized ZIF-62 (Co) using solvothermal method. C & D: SEM image of top surface (C) and cross-section (D) of freestanding $a_g$ZIF-62 (Co) membrane. Scale bar: 10 μm.

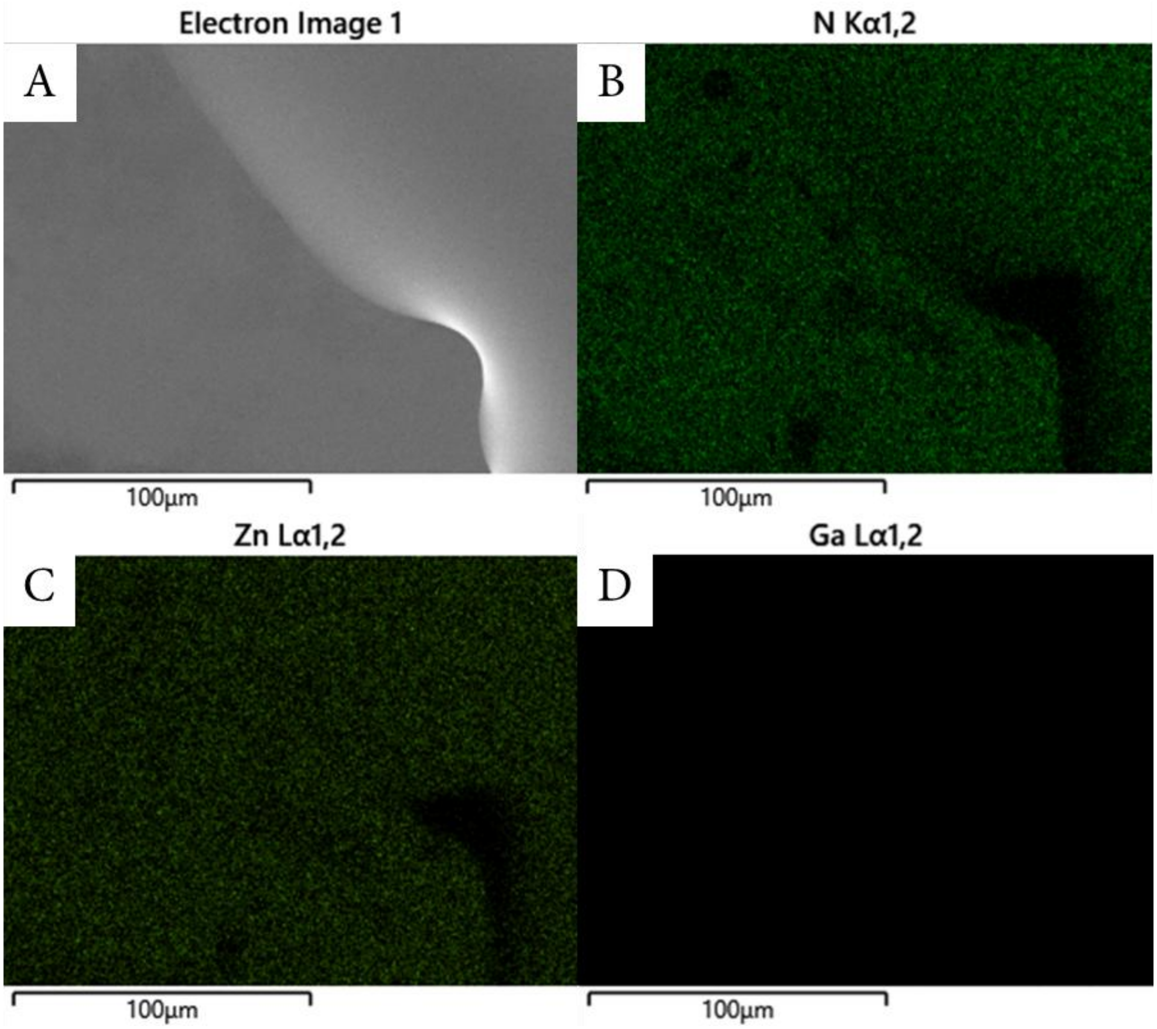


**Figure S3.** SEM image and EDS elemental maps of the top surface of a float glass processed $a_g$ZIF-62 membrane. A: Secondary electron image showing the membrane microstructure. B–D: Elemental mapping for nitrogen (N Kα), zinc (Zn Lα), and gallium (Ga Lα), respectively. The absence of detectable Ga signal in D suggests limited or no gallium incorporation within the probed region.

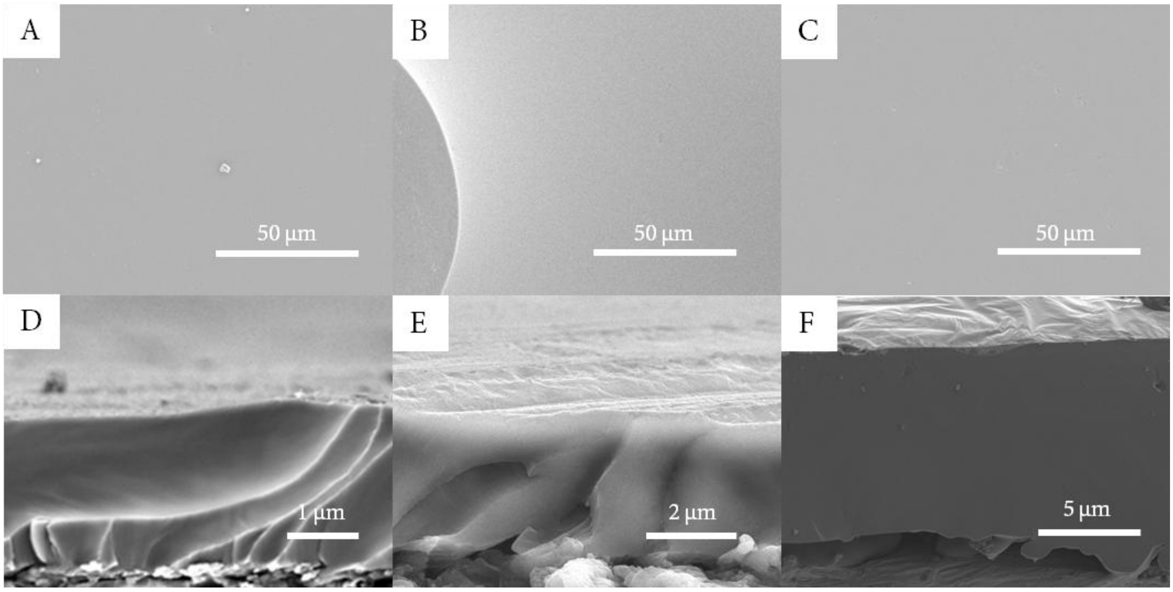


**Figure S4.** A–C: Top-view SEM images of agZIF-62 membranes prepared using 2.5 mg, 3.1 mg, and 8.8 mg of precursor material, respectively. D–F: Corresponding cross-sectional SEM images of the same membranes supported on carbon tape. Variations in cross-sectional texture arise from differences in sample preparation methods, including mechanical fracture (D&E) and blade-assisted cutting (F).

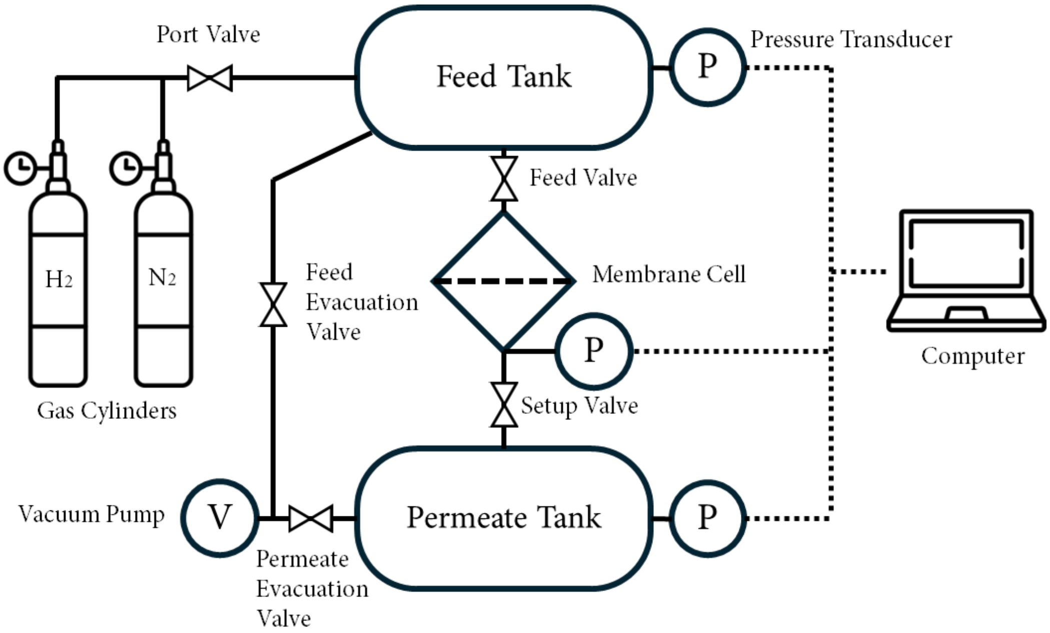


**Figure S5.** Schematic of the isometric gas permeation setup used for single-gas permeance measurements.

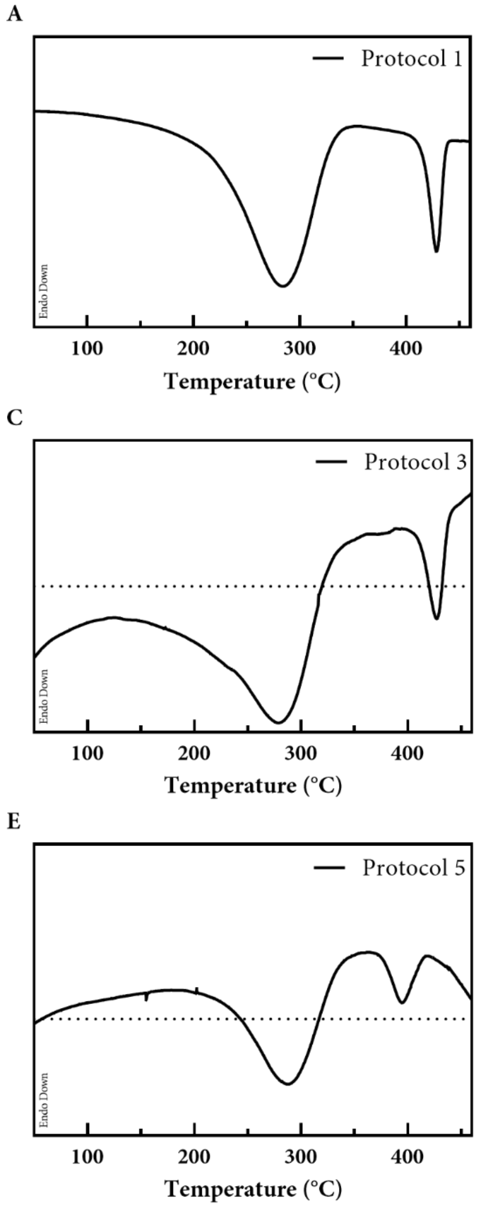


**Figure S6.** Differential scanning calorimetry (DSC) upscan curves of as-synthesized ZIF-62 crystals prepared using Protocols 1–6. The thermal profiles reveal characteristic endothermic transitions associated with the desolvation and melting and potential of the samples.

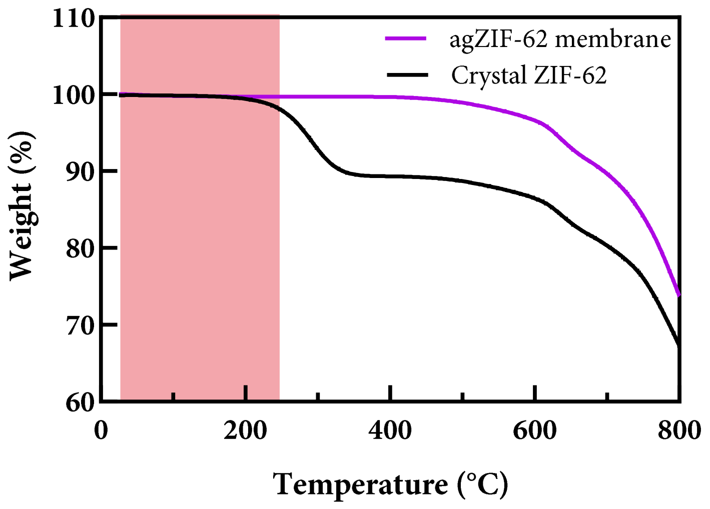


**Figure S7.** Thermogravimetric analysis (TGA) curves of agZIF-62 membrane and crystalline ZIF-62 under air atmosphere. The shaded red region indicates the temperature range used for high temperature gas permeation test.

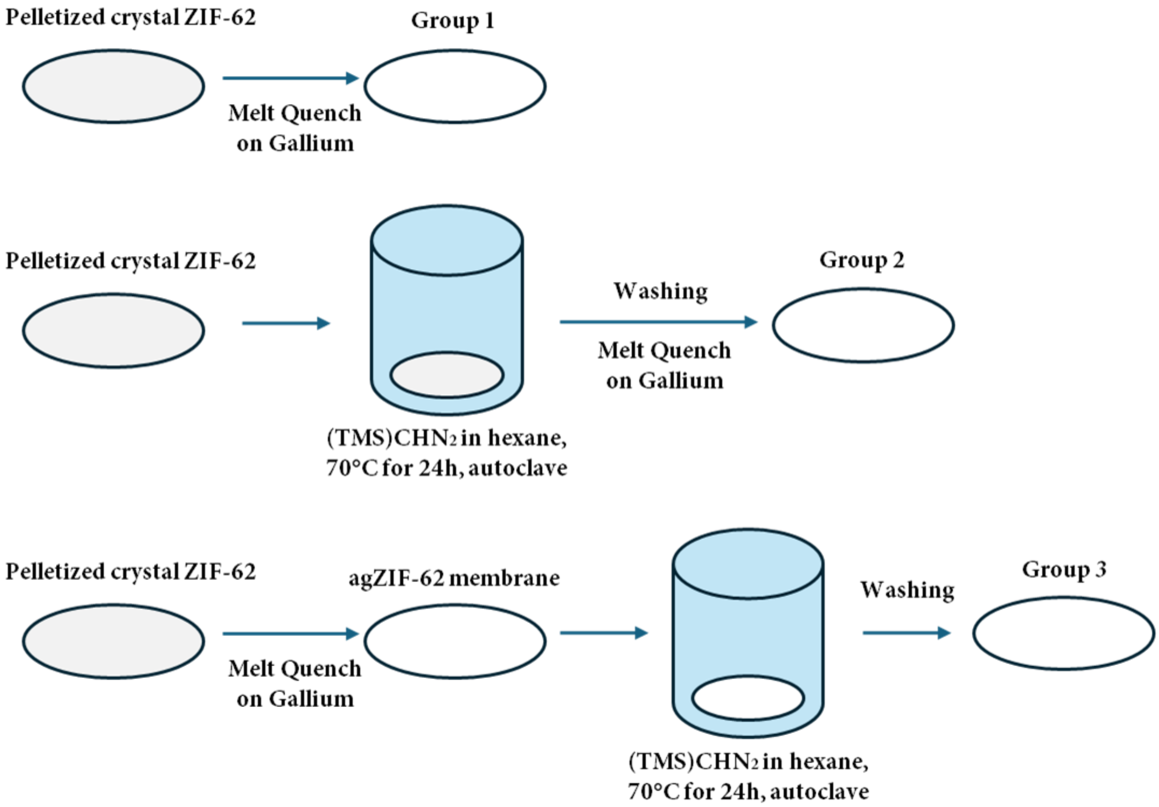


**Figure S8.** Schematic diagram showing fabrication of three types of glassy ZIF-62 membranes: untreated (Group 1), pre-treated crystals with (TMS)$CHN_2$ (Group 2), and post-treated amorphous membranes with (TMS)$CHN_2$ (Group 3).

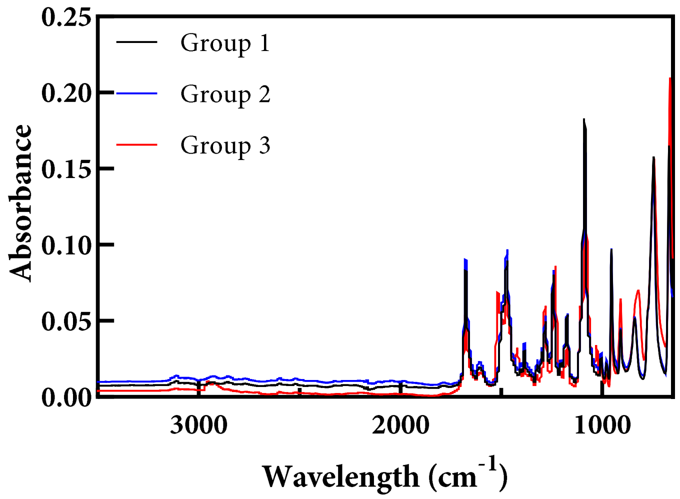


**Figure S9.** FTIR spectra of three types of glassy ZIF-62 membranes: untreated membranes (Group 1), membranes derived from (TMS)$CHN_2$-treated crystals (Group 2), and amorphous membranes post-treated with (TMS)$CHN_2$ (Group 3).

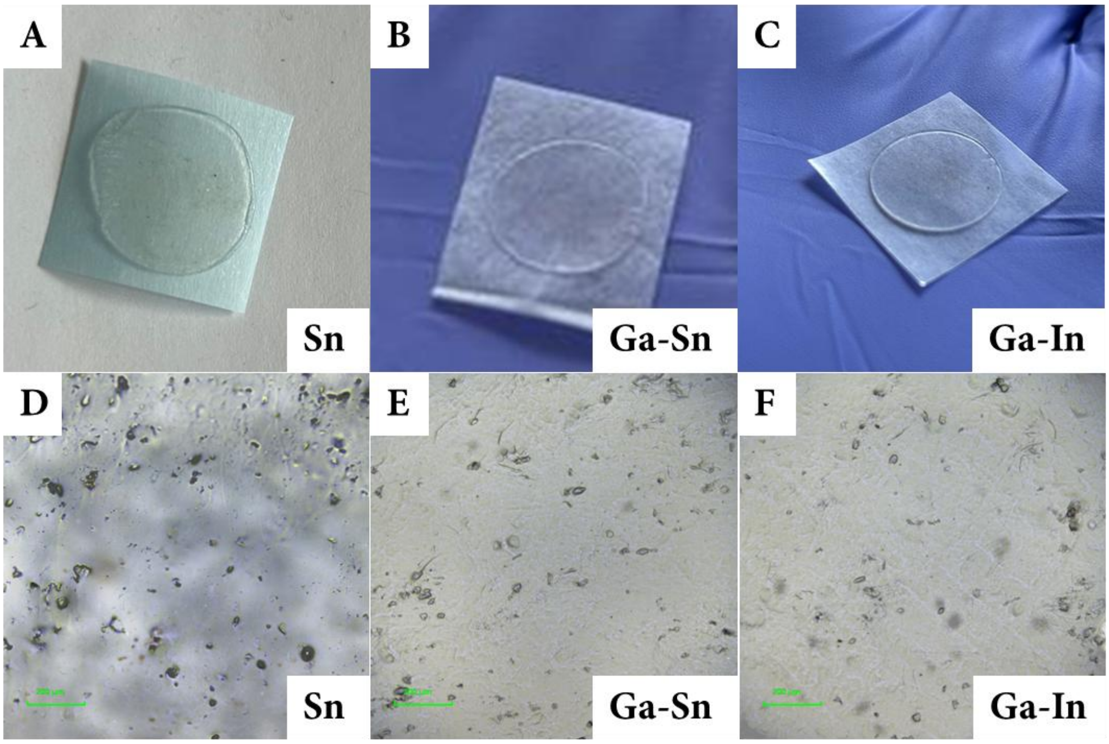


**Figure S10.** Optical (A–C) and laser profilometry (D–F) images of agZIF-62 membranes fabricated on different liquid metal substrates: Sn (A, D), Ga–Sn (10%) alloy (B, E), and Ga–In (25%) alloy (C, F).

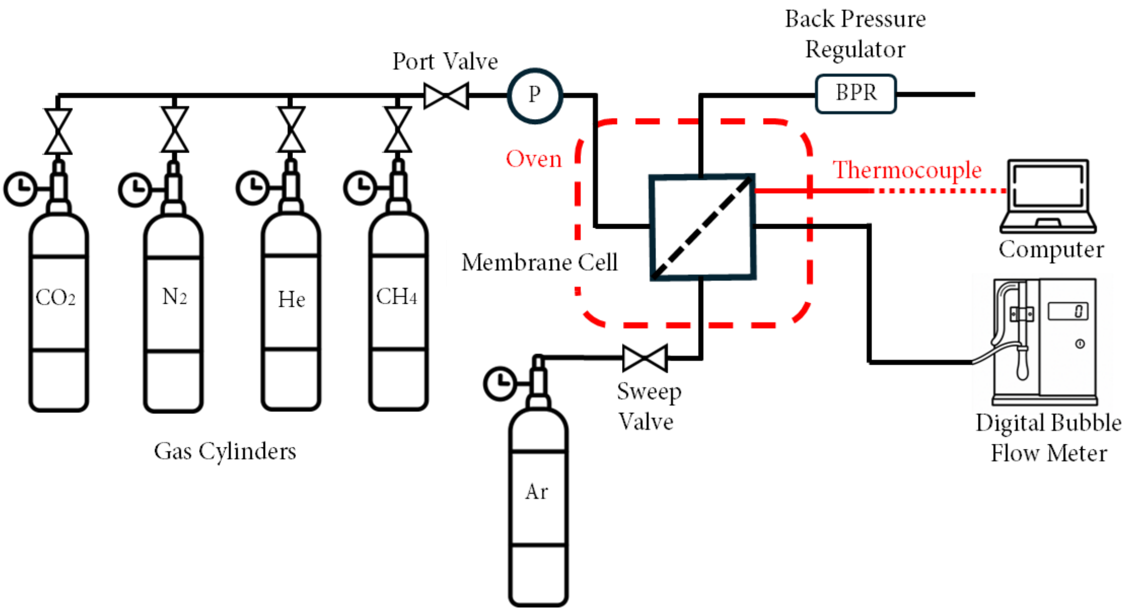


**Figure S11.** Isobaric gas permeation setup with integrated temperature control, used to measure gas permeance and apparent activation energies. A thermocouple is inserted into the membrane cell via a Swagelok fitting for precise temperature monitoring.

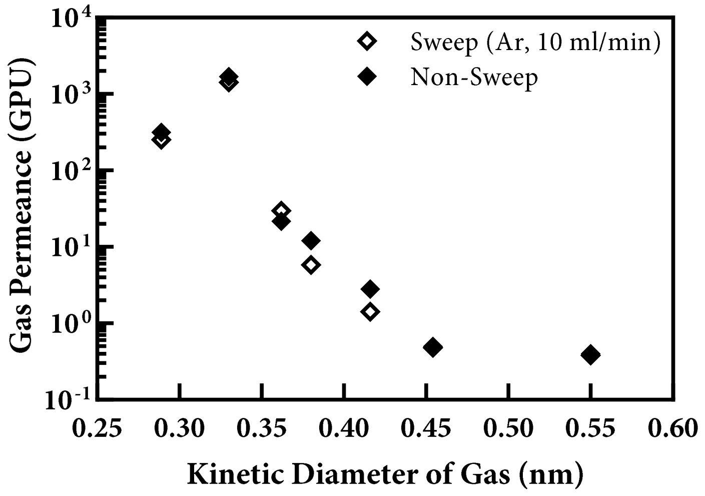


**Figure S12.** Effect of sweep gas on gas permeance in the isobaric gas permeation setup. Gas permeance is plotted as a function of kinetic diameter for various gases, comparing conditions with and without Ar sweep (10 mL/min). The use of sweep gas enhances the permeance of small gases by reducing concentration polarization and maintaining a higher driving force across the membrane.

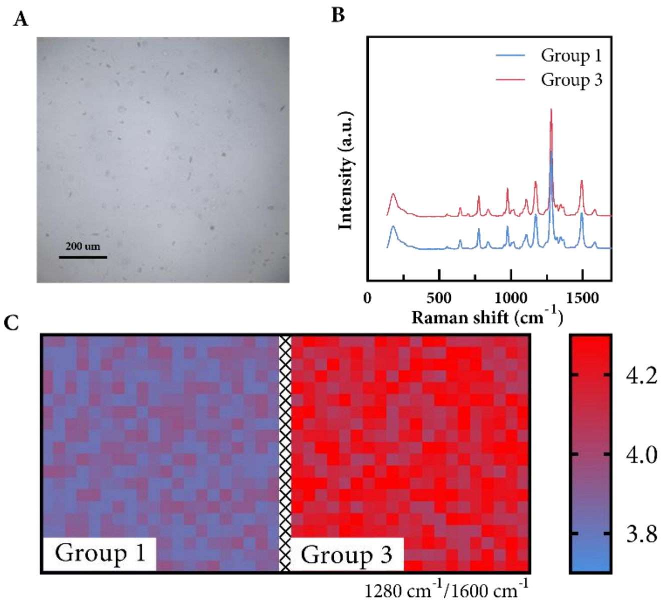


**Figure S13.** Raman analysis of untreated and methylated agZIF-62 membranes. (A) Optical image of the mapped untreated $a_g$ZIF-62 membrane surface (scale bar: 200 μm). (B) Representative Raman spectra from untreated (Group 1, blue) and methylated (Group 3, red) membranes. A distinct reduction of the C–N stretching peak at ~1280 $cm^{-1}$ is observed after methylation. (C) Raman intensity ratio mapping of 1280 $cm^{-1}$ (C–N) to 1600 $cm^{-1}$ (aromatic C=C) over a 20 × 20 grid of selected area.

## References

1. Nozari, V., Calahoo, C., Longley, L., Bennett, T. D. & Wondraczek, L. Structural integrity, meltability, and variability of thermal properties in the mixed-linker zeolitic imidazolate framework ZIF-62. *J. Chem. Phys.* **153**, 204501 (2020).
2. Smirnova, O. *et al.* Precise control over gas-transporting channels in zeolitic imidazolate framework glasses. *Nat. Mater.* **23**, 262–270 (2024).
3. Dickey, M. D. *et al.* Eutectic Gallium-Indium (EGaIn): A Liquid Metal Alloy for the Formation of Stable Structures in Microchannels at Room Temperature. *Adv. Funct. Mater.* **18**, 1097–1104 (2008).

4. König, U. & Keck, W. Measurement of the surface tension of gallium and indium in a hydrogen atmosphere by the sessile drop method. *J. Common Met.* **90**, 299–303 (1983).
5. White, D. W. G. The surface tensions of Pb, Sn, and Pb-Sn alloys. *Metall. Trans.* **2**, 3067–3071 (1971).
6. Yuan, Z. F. *et al.* Surface Tension and Its Temperature Coefficient of Molten Tin Determined with the Sessile Drop Method at Different Oxygen Partial Pressures. *J. Colloid Interface Sci.* **254**, 338–345 (2002).
7. Zhu, S., Lin, Q., Cao, R., Xie, K. & Lai, J. Interfacial Tension of Ga, E-GaIn, Galinstan, and GaInSnBiZn High-Entropy Alloy. *J. Mater. Eng. Perform.* **33**, 2369–2378 (2024).
8. Liu, T., Sen, P. & Kim, C.-J. Characterization of liquid-metal Galinstan® for droplet applications. in *2010 IEEE 23rd International Conference on Micro Electro Mechanical Systems (MEMS)* 560–563 (2010). doi:10.1109/MEMSYS.2010.5442440.
9. Dogan, A. & Arslan, H. Calculation of the surface tension of liquid Ga-based alloys. *Philos. Mag.* **98**, 1170–1185 (2018).
10. Qiao, A. *et al.* A metal-organic framework with ultrahigh glass-forming ability. *Sci. Adv.* **4**, eaao6827 (2018).
11. Hou, J. *et al.* Halogenated Metal–Organic Framework Glasses and Liquids. *J. Am. Chem. Soc.* **142**, 3880–3890 (2020).
12. Freeman, B. D. Basis of Permeability/Selectivity Tradeoff Relations in Polymeric Gas Separation Membranes. *Macromolecules* **32**, 375–380 (1999).
13. Thornton, A. W. *et al.* Analytical representation of micropores for predicting gas adsorption in porous materials. *Microporous Mesoporous Mater.* **167**, 188–197 (2013).
